# Book Notices

**Published:** 1888-07

**Authors:** 


### BOOK NOTICES.

THE HYGIENE OF THE SKIN; OR THE ART OF PREVENTING SKIN DISEASES. By A. Ravoyli, M. D., 400 pages; Cincinnati; Central Medical Publishing Company, 1888.

This volume is neatly printed in clear type, and has a number of illustrations scattered through it, representing leprosy, poison sumach; the dry mercurial bath, etc. If one has time to read the book he will find plenty that will instruct him and will be of practical value to him in every day life. But the book is a curious medley of information, intended, it seems, to occupy the medium ground between the profession and the laity. It is sufficiently scientific and practical to deserve a place in every man's library whether he is a physician or layman.

---

ATLAS OF VENEREAL AND SKIN DISEASES, BY PRINCE A. MORROW, A. M., M. D., Clinical Professor of Venereal Diseases, formerly Clinical Lecturer on Dermatology in the University of the City of New York, etc. Published by William Wood & Co., New York.

This Atlas is an imperial folio, and will consist of fifteen parts, containing, in all, seventy-five chromo-lithographic plates, and several hundred figures, many of them life size, in flesh tints and colors, together with a descriptive text for each plate, and from sixteen ro twenty folio pages of a practical treatise upon venereal and skin diseases; the whole, after all the parts are issued, to form one volume. Parts I, II, III, IV and V are before us. The price of each is $2. Sold only on subscription. The advantage of these fasciculi over the ordinary text book, is very great. The busy practitioner can not only read in a few minutes what he wants, but he can see it in life size and appearance.

The author is so well known, through other works of his, that it is unnecessary to do more than mention his connection with the Atlas. Professor Morrow and the publishers have done themselves great credit in bringing out this, the largest, the best arranged, and most complete work on these subjects we have ever had the pleasure of examining. No physician's library should be considered complete without Morrow's Atlas of Venereal and Skin diseases.

We look forward with special pleasure to the issuing of the other ten numbers of this valuable Atlas, which have been promised this year.

Publisher Geo. S. Davis, of Detroit, Mich., is getting out monthly a handsome pamphlet entitled, "The Physician's Leisure Library. No. 7 is before us, containing the modern treatment of pleurisy and pneumonia, by G. M. Garland, M. D. of the Harvard Medical School. This is an innovation in medical literature, and judging from the number before us the plan should grow into great popularity.

---

A REFERENCE HANDBOOK OF THE MEDICAL SCIENCES.—Embracing the entire range of Scientific and Practical Medicine and Allied Science. Volume VI., by various writers. Illustrated by chromo-lithographs and fine wood engravings. Edited by ALBERT H. BUCK, M. D. Complete in eight volumes. Price per vol., muslin, $6.00; sheep, $7.00; half morocco, $8.00. NEW YORK: WILLIAM WOOD AND COMPANY.

This volume is a most valuable book, and adds greatly to the series. The handsome full page colored plate of the horrid *Crotillus,* the diamond rattlesnake, and ditto of the "*Gila Monster*"—*Hyloderma suspectus*, or poisonous lizzard of Mexico, together with the admirable chapter accompanying them on "Venomous Reptiles," by Dr. H. Creecy Yarrow, Superintendent of Department of Reptiles, National Museum, Washington, are alone worth the price of the entire series. There is also a full page colored plate representing a case of tubercular syphilide which is very fine. Altogether this is a monumental work of great value, and every progressive physician and scientist should avail himself of the rare opportunity here presented of enriching his library at a small cost. Wood & Co., New York, are the caterers to the æsthetic taste of medical men.

